# Suture-and-clip continuous suturing technique: a novel endoscopic closure method for large mucosal defects

**DOI:** 10.1055/a-2695-4279

**Published:** 2025-11-05

**Authors:** Lingyu Zhu, Yongshuai Liu, Shengquan Lin, Xiaodong Zhong, Hongmei Qu, Shanming Sun, Shilin Qiu

**Affiliations:** 1 117907 Department of Gastroenterology, Weifang Peopleʼs Hospital, Weifang, China; 2 Department of Anesthesiology, Weifang Hospital of Traditional Chinese Medicine, Weifang, China

We describe a case of a 56-year-old man in whom upper endoscopy revealed early gastric cancer at the greater curvature of the lower gastric body ( Fig. 1 **a** ). Endoscopic submucosal dissection (ESD) was performed to achieve complete resection of the lesion. Following resection, a 40 × 35-mm mucosal defect remained ( Fig. 1 **b** ). To prevent delayed perforation and bleeding, the defect required closure. As the gastric wall thickness and lesion tension precluded approximation using clips alone, we employed a novel suturing strategy utilizing surgical suture material.

**Fig. 1:**
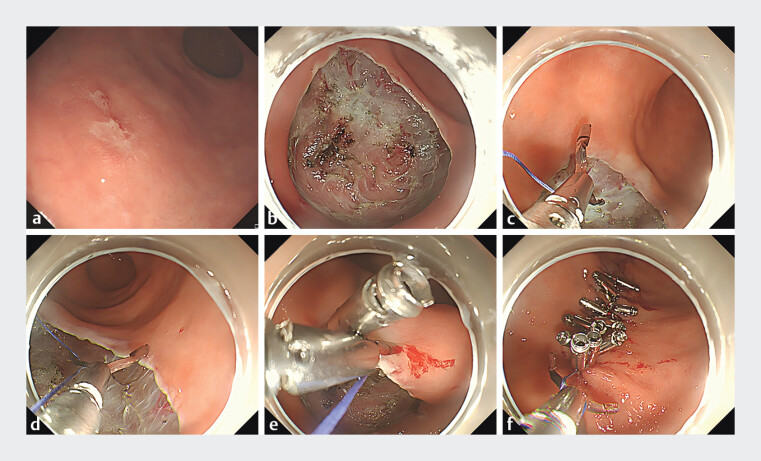
Endoscopic views of the closure of a large gastric mucosal defect after endoscopic submucosal dissection (ESD). **a** Early gastric cancer at greater curvature of lower gastric body. **b** A 40 × 35-mm mucosal defect post-ESD. **c** A reopenable clip with sutures was placed on the distal edge of the defect. **d** A second clip was hooked onto the suture and placed on the opposite side of the defect. **e** The suture was pulled to tighten and close the defect. **f** The last clip was hooked onto the suture, rotated 360 degrees, and placed at the end of the defect.

The procedure for defect closure was as follows ( Video 1 ). An absorbable surgical suture was secured to one arm of a reopenable clip. The first clip (with attached suture) was deployed at the distal margin of the defect, engaging the full thickness of the gastric wall ( Fig. 1 **c** ). Thereafter, a second clip was hooked onto the suture and placed on the opposite side of the defect ( Fig. 1 **d** ). The suture was pulled to approximate the edges, with sequential repetition of this process until achieving complete tension-free closure ( Fig. 1 **e** ). Ultimately, reinforcement clips were placed to consolidate the closure. For the terminal clip, it was hooked onto the suture, rotated 360°, and anchored at the proximal defect margin ( Fig. 1 **f** ). Finally, the suture was transected using a hemostat. Gastroscopy performed one week later did not reveal a split defect or detachment of the clip ( Fig. 2 ).

Novel endoscopic closure method for large mucosal defects.Video 1

**Fig. 2:**
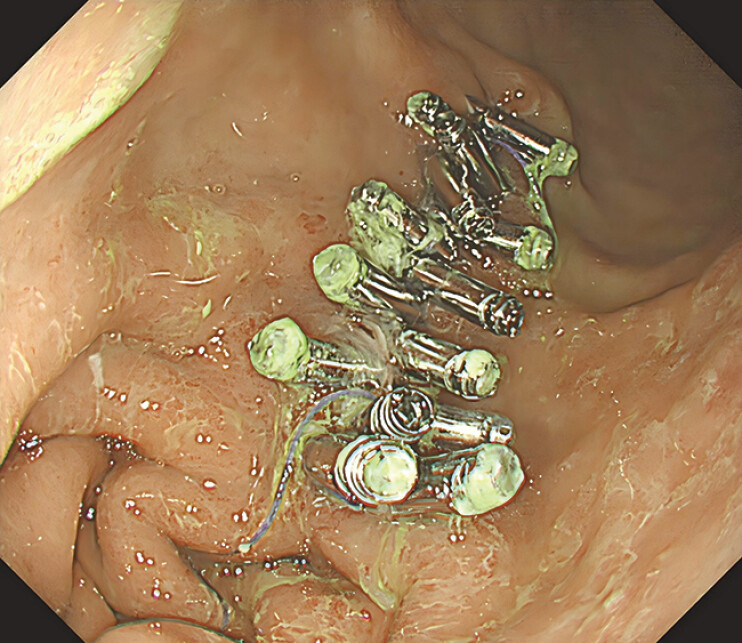
Gastroscopy performed one week later did not reveal a split defect or detachment of the clip.

Large post-ESD mucosal defects may lead to serious postoperative complications, including delayed bleeding and perforation 1 . Although novel endoscopic closure techniques have emerged, most require specialized devices with significant technical complexity 2 3 . Our technique utilizes universally available absorbable sutures combined with standard clips to achieve continuous endoscopic suturing. This method provides rapid, accessible closure with balanced tension distribution, representing a potentially valuable advancement in endoscopic defect management.

Endoscopy_UCTN_Code_CPL_1AJ_2AJ

## Notes

Endoscopy E-Videos https://eref.thieme.de/e-videos
*E-Videos* is an open access online section of the journal *Endoscopy* , reporting on interesting cases and new techniques in gastroenterological endoscopy. All papers include a high-quality video and are published with a Creative Commons CC-BY license. Endoscopy E-Videos qualify for HINARI discounts and waivers and eligibility is automatically checked during the submission process. We grant 100% waivers to articles whose corresponding authors are based in Group A countries and 50% waivers to those who are based in Group B countries as classified by Research4Life (see: https://www.research4life.org/access/eligibility/ ). This section has its own submission website at https://mc.manuscriptcentral.com/e-videos .
